# Reappraising the role of the vagus nerve in GLP‐1‐mediated regulation of eating

**DOI:** 10.1111/bph.15603

**Published:** 2021-07-31

**Authors:** Daniel I. Brierley, Guillaume de Lartigue

**Affiliations:** ^1^ Centre for Cardiovascular and Metabolic Neuroscience, Department of Neuroscience, Physiology and Pharmacology University College London London UK; ^2^ Center for Integrative Cardiovascular and Metabolic Disease University of Florida Gainesville Florida USA

**Keywords:** eating, feeding, glucagon‐like peptide‐1, neuropeptide, obesity, preproglucagon, vagus nerve

## Abstract

Here, we provide a focused review of the evidence for the roles of the vagus nerve in mediating the regulatory effects of peripherally and centrally produced GLP‐1 on eating behaviour and energy balance. We particularly focus on recent studies which have used selective genetic, viral, and transcriptomic approaches to provide important insights into the anatomical and functional organisation of GLP‐1‐mediated gut‐brain signalling pathways. A number of these studies have challenged canonical ideas of how GLP‐1 acts in the periphery and the brain to regulate eating behaviour, with important implications for the development of pharmacological treatments for obesity.

**LINKED ARTICLES:**

This article is part of a themed issue on GLP1 receptor ligands (BJP 75th Anniversary). To view the other articles in this section visit http://onlinelibrary.wiley.com/doi/10.1111/bph.v179.4/issuetoc

**Abbreviations**
FFA: free fatty acid
GLP‐1: glucagon‐like peptide 1
NTS: nucleus tractus solitarius
PPG: preproglucagon
PVN: paraventricular nucleus of the hypothalamus
SGLT1: sodium‐dependent glucose transporter 1

## 1 INTRODUCTION

The prevalence of obesity is increasing at an alarming rate. This chronic relapsing disease is associated with co‐morbidities including type 2 diabetes, cardiovascular disease, stroke and cancer, resulting in higher mortality rates amongst individuals living with obesity (Abdelaal et al., 2017; Bray et al., 2017). Although there is a consensus that increased energy intake, coupled with reduced energy expenditure, is the primary impetus for weight gain, lifestyle interventions aimed at reducing food intake and increasing exercise are largely ineffective in treating obesity (Grill, 2020). It appears that physiological mechanisms that prevent weight gain fail during the initial onset of obesity, yet defend against weight loss once obesity has developed. Although much attention has been given to the role of the brain in energy homeostasis, at present, remodelling of the gut through bariatric surgery is the only effective treatment for severe obesity. This implicates neuronal and/or hormonal gut‐brain signalling mechanisms in the development of obesity, and suggests that targeting these mechanisms provides the most effective opportunities for obesity pharmacotherapy.

Glucagon‐like peptide 1 (GLP‐1), a gastrointestinal hormone and centrally produced neuromodulator, has received particular attention as a gut‐brain signalling molecule that can be targeted for the treatment of metabolic diseases. GLP‐1 is predominantly released from the gut in response to nutrient ingestion, with known roles in glucose homeostasis, gut motility and the regulation of eating. In humans and rodents, GLP‐1 levels are reduced in obesity, while bariatric surgery dramatically increases post‐prandial levels of GLP‐1 (Chambers et al., 2014; Jiménez et al., 2014; Umeda et al., 2011) as early as a few days after the surgery (Le Roux et al., 2007). The level of circulating GLP‐1 after bariatric surgery positively correlates with both improved insulin release (Umeda et al., 2011) and weight loss (Le Roux et al., 2007), while administration of the GLP‐1 receptor antagonist exendin‐(9‐39) blocks the surgery‐induced insulin response to an oral glucose load in both humans and rodents (Chambers et al., 2011; Salehi et al., 2011; Vidal et al., 2016). In line with the pleiotropic effects of GLP‐1, pharmacological therapies aimed at increasing GLP‐1 levels by stimulating GLP‐1 secretion (Iwasaki et al., 2018; Vidal et al., 2016), inhibiting proteolytic breakdown of GLP‐1 (Herman et al., 2006), or exogenous administration of GLP‐1 analogues (Vilsbøll et al., 2012), are effective in promoting weight loss and improve a number of obesity‐associated health risks (Campbell & Drucker, 2013; Kim et al., 2013).

GLP‐1 is also produced centrally by neurons in the caudal brainstem, a key integration site for gut‐brain signalling pathways which regulate energy homeostasis, particularly those mediated by the vagus nerve (Brierley et al., 2021; Holt, Richards, et al., 2019; Llewellyn‐Smith et al., 2011; McLean et al., 2020). Here, we critically review evidence for the role(s) of the vagus nerve in mediating the regulatory effects of peripherally and centrally produced GLP‐1 on eating behaviour and metabolism. We particularly highlight recent studies which have used selective genetic and viral approaches to provide important insights into the anatomical and functional organisation of GLP‐1‐mediated gut‐brain signalling pathways. We discuss how these recent findings challenge canonical views of how GLP‐1 regulates eating, and the implications of these findings for the development of pharmacological obesity treatments.

## 2 VAGAL‐DEPENDENT ACTIONS OF GLP‐1 IN THE PERIPHERY

### 2.1 GLP‐1 production in the gut

Circulating GLP‐1 levels are low under fasting conditions and increase threefold to fourfold in response to a meal (Ørskov et al., 1996). Consumption of food high in fats and/or carbohydrates is the primary physiological stimulus for GLP‐1 secretion (Brubaker, 2006; Herrmann et al., 1995). This secretion is predominantly mediated by nutrient sensing in the gut, since direct infusion of individual macronutrients into the intestine is sufficient to increase circulating GLP‐1 levels (Cordier‐Bussat et al., 1998; Roberge & Brubaker, 1993; Rocca & Brubaker, 1999). Furthermore, orally consumed glucose elicits a rapid rise in GLP‐1, however, bypassing the gastrointestinal tract with intravenous infusion of glucose is not sufficient to elicit this rise (Herrmann et al., 1995; Unger et al., 1968). Nutrient sensing in the gut is performed by specialised cells known as enteroendocrine cells, which are sparsely dispersed in the gastrointestinal epithelium. Enteroendocrine cells sense luminal content via long projections into the gut lumen and/or via post‐absorptive mechanisms, resulting in the release of hormones that are produced and packaged into dense core vesicles, through a process of regulated exocytosis. L‐cells are enteroendocrine cells which express the glucagon gene and prohormone convertase 1/3 enzyme, necessary for post‐translational GLP‐1 production, and account for 95% of circulating GLP‐1 (Song et al., 2019).

L‐cells are distributed in an increasing gradient along the length of the gastrointestinal tract, occurring at low frequency in the duodenum, increasing in the jejunum and peaking in the ileum and colon (Gribble & Reimann, 2021; Sjolund et al., 1983). GLP‐1 immunoreactive cells are also located in the gastric mucosa, although in low numbers, and these gastric GLP‐1 producing cells are capable of transiently releasing GLP‐1 in response to dietary intake (Ribeiro‐Parenti et al., 2021). Interestingly, increased L cell density and GLP‐1 concentration per volume of tissue in the more distal gut is well conserved across species (Kuhre et al., 2014). At least in mice, anatomically segregated L‐cell populations co‐express different complements of hormones, suggesting that proximal and distal L‐cells may serve different functions (Paternoster & Falasca, 2018). In rodents, there is evidence that GLP‐1 is released in two phases: an early phase that starts within minutes of nutrient ingestion; and a late phase starting 30–60 min after food intake (Elliott et al., 1993; Herrmann et al., 1995). This biphasic release pattern has been thought to reflect activation of separate populations of L‐cells as nutrients progress through the gastrointestinal tract. In support of this hypothesis, deletion of the glucagon gene in both proximal and distal L‐cells in mice abolished both the early and late phases of nutrient‐induced GLP‐1 secretion. However, deletion of the same gene selectively in distal L‐cells had no effect (Panaro et al., 2020). This suggests that, contrary to conventional dogma, proximal L‐cells are involved in both early and late phase GLP‐1 release, via mechanisms that involve direct nutrient sensing and/or rapid cephalic‐phase neuronal control, while distal L‐cells may play a prominent role in the “ileal break”, a potent negative feedback mechanism for slowing down intestinal transit in response to elevated undigested nutrient levels in the ileum (Maljaars et al., 2008). However, the fact that distal GLP‐1 L‐cells are dispensable for GLP‐1 secretion in response to fats and proteins raises questions about their normal physiological role in eating (Panaro et al., 2020). Instead, distal L‐cells are responsive to bacterial metabolites, which increase exponentially along the length of the gut and are particularly sensitive to pharmacological therapeutics (Panaro et al., 2020). Clarifying the role of the distal L‐cells in models of bariatric surgery will be important. In humans, a longer monophasic GLP‐1 release is more commonly reported (Vollmer et al., 2008). Whether this reflects different mechanisms of release between species, or if the larger capacity of human L cells prolongs GLP‐1 release, resulting in the merge of the two phases, is an important distinction that has not yet been resolved.

### 2.2 Nutrient‐induced GLP‐1 release

L‐cells are electrically excitable and release GLP‐1 via a process of regulated exocytosis following receptor and/or ion channel activation. Calcium‐ and cAMP‐dependent intracellular mechanisms are activated by a variety of nutritional, neuronal and hormonal factors. Dietary carbohydrates and lipids are particularly potent stimulators of intestinal L‐cells. Glucose is detected by proximal L‐cells via the sodium/glucose transporter 1 (SGLT1) expressed on the luminal side of L‐cells. The influx of sodium ions depolarizes the L‐cell, triggering the release of GLP‐1 (Powell et al., 2013). The use of nonspecific SGLT inhibitors, or knockout mice lacking SGLT1, abolishes early phase GLP‐1 release mediated by proximal L‐cells (Gorboulev et al., 2012; Sun et al., 2017). Interestingly, the reduced glucose absorption associated with pharmacological or genetic inhibition of SGLT1 results in a sizeable second phase GLP‐1 release, suggesting that increased glucose transit activates distal L‐cells and releases GLP‐1 via a SGLT1‐independent mechanism (Powell et al., 2013). In support of this, increased distal glucose delivery following bariatric surgery increases GLP‐1 levels independent of SGLT1 (Martinussen et al., 2020). Three alternative mechanisms for glucose‐induced GLP‐1 secretion from L‐cells have been proposed: (1) closure of ATP‐sensitive potassium channels in response to ATP elevation after the metabolism of glucose; (2) activation of G protein‐coupled sweet taste receptors (TAS1R1 and TAS1R3) expressed on colonic L‐cells; and (3) activation of L‐cells by a microbial factor after glucose fermentation by microbiota in the distal intestine (Buckley et al., 2020).

In humans, high protein meals (Lejeune et al., 2006; Van Der Klaauw et al., 2013) and peptide hydrolysates (Calbet & Holst, 2004) increase circulating GLP‐1 levels. In cell lines, a variety of individual amino acids were sufficient for GLP‐1 release (Gameiro et al., 2005; Reimann et al., 2004; Tolhurst et al., 2011). At the molecular level, protein sensing by the L‐cell involves the calcium sensing receptor (CaSR) and the peptide transporter 1 (PEPT1), with calcium being required for protein‐induced GLP‐1 secretion (Diakogiannaki et al., 2013). Dietary fats are also potent GLP‐1 secretagogues (Feltrin et al., 2004). Blocking fat digestion with lipase inhibitors blunts GLP‐1 release after a fatty meal (Beglinger et al., 2010; Ellrichmann et al., 2008), suggesting that the breakdown products of lipids are necessary. L‐cells express G‐protein coupled receptors that sense a range of different fatty acids (FFAR1‐4; Bolognini et al., 2016; Edfalk et al., 2008) and monoglycerides (GPR119; Hansen et al., 2011). Fat induced GLP‐1 secretion is blunted in knockout mice lacking FFA1 (Xiong et al., 2013), FFA2 (Tolhurst et al., 2012), FFA3 (Tolhurst et al., 2012), and GPR119 receptors (Moss et al., 2016), with no change in GLP‐1 levels reported in *Ffar4* null mice (Xiong et al., 2013). Both Gq‐ and Gs‐dependent intracellular signalling mechanisms have been implicated in fat mediated GLP‐1 secretion (Hauge et al., 2016). Interestingly, L‐cells may express FFA receptors on their basolateral membrane and sense fatty acids that are absorbed across the epithelium, rather than directly in the lumen. In isolated intestinal preparations, long‐chain fatty acids, FFA1 agonists (Christensen et al., 2015), or short‐chain fatty acid infusions (Christiansen et al., 2018) into the vasculature, but not the lumen, increase GLP‐1 levels. Furthermore, GLP‐1 release is activated by chylomicrons, the breakdown products of lipid digestion, which are packaged into triglyceride‐rich lipoproteins by enterocytes and released onto the basolateral side (Psichas et al., 2017).

In addition to nutrient stimuli, GLP‐1 release can also be modulated by neural and endocrine mechanisms. Stimulating the celiac branch of the vagus nerve in rats (Rocca & Brubaker, 1999), or administration of muscarinic receptor agonists to mimic parasympathetic activity in primary L‐cell cultures (Anini et al., 2002; Anini & Brubaker, 2003), both promote GLP‐1 release, implicating the parasympathetic nervous system in control of GLP‐1 release. Conversely, surgical lesioning of the subdiaphragmatic vagus nerve, or administration of muscarinic receptor antagonists, prevents early phase GLP‐1 release in response to intestinal glucose (Balks et al., 1997), and both first and second phase GLP‐1 secretion in response to intestinal infusion of large volumes of fat (Anini et al., 2002). These data suggest the possibility that GLP‐1 release from L‐cells can be triggered by top‐down neural mechanisms. Furthermore, several circulating hormones and neuropeptides are able to modulate GLP‐1 secretion. Leptin (Anini & Brubaker, 2003), glucose‐dependent insulinotropic peptide (GIP) (Roberge & Brubaker, 1993), and gastrin‐releasing peptide (GRP) (Persson et al., 2000), increase GLP‐1 release, while insulin (Lim et al., 2009) and somatostatin (Moss et al., 2012) provide negative feedback, demonstrating that GLP‐1 release from the gut is under multiple levels of control.

### 2.3 GLP‐1 site of action

A comprehensive assessment of the physiological role(s) of gut‐derived GLP‐1 has not yet been completed, but intraperitoneal injection of GLP‐1 has been shown to advance satiation, inhibit gastric emptying and improve glucose tolerance (Krieger et al., 2016). Three separate strategies have been recently employed to assess the physiological role of gut‐derived GLP‐1. One genetic strategy deleted the glucagon gene in intestinal epithelial cells, to selectively prevent GLP‐1 production within intestinal L‐cells (Panaro et al., 2020; Song et al., 2019). These mice had impaired oral glucose tolerance (Song et al., 2019), but cumulative food intake and bodyweight were unaffected (Panaro et al., 2020), although neither gastric emptying nor satiation were explicitly tested. A separate genetic strategy expressed Gq‐coupled designer receptors exclusively activated by designer drugs (DREADD) under the control of the *Insl5* promoter, to allow chemogenetic stimulation of colonic L‐cells (Lewis et al., 2020). Stimulation of L‐cells with the designer drug clozapine *N*‐oxide (CNO) improved glucose tolerance and significantly reduced food intake and body weight, although the latter effects may have been mediated by peptide YY (PYY), which is co‐secreted with GLP‐1 from L‐cells. Finally, oral administration of the non‐nutritive sweetener, D‐allulose, was found to selectively increase active GLP‐1 in the hepatic portal vein without altering the levels of other gut hormones (Iwasaki et al., 2018). Oral, but not intravenous, administration of D‐allulose dose‐dependently decreased short‐term food intake and improved insulin‐mediated glucose tolerance. Thus, the release of GLP‐1 from L‐cells is both necessary and sufficient for glucose tolerance, sufficient for short‐term control of food intake, and has an as yet unclear role in gastric emptying.

The actions of GLP‐1 are mediated by a single class B GPCR, the GLP‐1 receptor (Thorens, 1992). As would be expected, mice in which the GLP‐1 receptor gene *(Glp1r*) has been globally deleted have impaired gastric emptying, elevated fasting glucose, and a blunted insulin response to oral glucose (Scrocchi et al., 1996). Furthermore, the effect of i.p. GLP‐1 or oral administration of D‐allulose on glucose clearance are completely abolished in these mice. Surprisingly, these *Glpr1r* ^*‐/‐*^ null mice display normal body weight, overall food intake and energy expenditure. However, the satiating effects of D‐allulose (Iwasaki et al., 2018), as well as peripheral (Baggio et al., 2004) or central (Scrocchi et al., 1996) injections of GLP‐1 are all abolished in these mice. Many of these outcomes have been replicated with the GLP‐1 antagonist, exendin‐(9–39) (Williams et al., 2009). Together, these data suggest that GLP‐1 receptors are sufficient to mediate all the effects of GLP‐1, but the effects of endogenous GLP‐1 on eating behaviour are: (1) compensated for in the knockout mice by functionally overlapping/redundant mechanisms; and/or (2) have little impact on long‐term energy homeostasis under *ad libitum* feeding conditions in laboratory mice.

GLP‐1 receptors are widely distributed throughout the periphery (Bullock et al., 1996; Nakagawa et al., 2004; Vahl et al., 2007) and brain (Cork et al., 2015; Larsen et al., 1997; Merchenthaler et al., 1999). Central administration of a GLP‐1 antagonist increases food intake, demonstrating that endogenous GLP‐1 regulates feeding behaviour through its actions on central GLP‐1 receptors (Turton et al., 1996). Activation of central GLP‐1 receptor‐expressing neuronal populations using pharmacological or genetic approaches indicates that GLP‐1 recruits both homeostatic and hedonic feeding circuits (Li et al., 2019; McLean et al., 2020; Turton et al., 1996). Nevertheless, evidence is mounting against a primary role for central GLP‐1 receptors in mediating the physiological effects of endogenous peripheral GLP‐1 released from L‐cells in response to a meal. Firstly, GLP‐1 in the circulation has a half‐life of <2 min, so this rapid degradation suggests that hormonal GLP‐1 may not reach physiologically relevant levels in the circulation, and therefore must primarily act via paracrine mechanisms within the gut. The enzyme responsible for GLP‐1 degradation, dipeptidyl‐peptidase 4, is widely distributed, including in soluble form in the circulation (Mentlein, 1999), and bound to epithelial cells lining the hepatic portal vein (Hansen et al., 1999) and in the liver (Mentzel et al., 1996). More than half of GLP‐1 secreted in response to a meal is inactivated by the time it reaches the hepatic portal vein (Hansen et al., 1999) and less than 20% of active GLP‐1 exits the liver (Ruttimann, 2010). Secondly, i.p. GLP‐1‐induced satiation is not impaired by central injections of the GLP‐1 antagonist exendin‐(9–39). However, peripheral exendin‐(9–39) completely abolishes the anorexigenic effect of i.p. GLP‐1 and inhibits sugar intake (Williams et al., 2009). Finally, conditional deletion of GLP‐1 in CNS neurons using a *Nes*‐Cre mouse (Sisley et al., 2014) or in both CNS and enteric neurons with a *Wnt1*‐Cre mouse (Varin et al., 2019), has no effect on glucose tolerance, gastric emptying or food intake. These findings argue against gut‐derived GLP‐1 reaching the CNS to directly regulate eating behaviours, at least under *ad libitum* feeding conditions in laboratory rodents.

Peripheral administration of the GLP‐1 agonists exendin‐4, liraglutide, and semaglutide, which are resistant to rapid dipeptidyl peptidase 4 degradation and so have half‐lives extended by hours, potently reduce food intake via central mechanisms (Almandoz et al., 2020; Hayes, Kanoski, Alhadeff, & Grill, 2011). The anorexigenic effect of peripheral administration of GLP‐1 agonists is abolished in mice with CNS‐specific deletion of GLP‐1 receptor (Sisley et al., 2014) and following central administration of a GLP‐1 antagonist (Barrera et al., 2011). Deletion of GLP‐1 receptors on autonomic neurons under the control of a *Phox2b* promoter has no effect on liraglutide induced inhibition of food intake, body weight or glucose homeostasis (Sisley et al., 2014). However, the beneficial effect of more peripherally restricted GLP‐1 agonists such as albiglutide and dulaglutide is partially impaired in this mouse line (Varin et al., 2019). These data support the idea that endogenous GLP‐1 and GLP‐1 agonists mediate their effects via different mechanisms. Importantly, this suggests that under conditions where it avoids degradation by DDP‐IV, peripheral GLP‐1 can access central GLP‐1 receptor populations in the circumventricular organs, which are outside the blood brain barrier (Gabery et al., 2020; Secher et al., 2014). In support of this, supraphysiological doses of GLP‐1 injected directly into the hepatic portal vein suppress eating through a central mechanism that requires hindbrain GLP‐1 receptors and an intact area postrema (Punjabi et al., 2014). Importantly, this only occurs with supraphysiological GLP‐1 concentrations that are 4–5 times higher than those reported after meals. Such elevated levels can be reached in animals that have undergone bariatric surgery (Peterli et al., 2012), as a result of the increased unabsorbed nutrients reaching the distal L‐cells (Larraufie et al., 2019) and/or reduced dipeptidyl peptidase 4 expression (Herz et al., 2021).

### 2.4 Role for vagal sensory neurons in intestinal GLP‐1 signalling

The evidence described above strongly suggests that intestinal GLP‐1 predominantly produces its effects through a neuronally mediated paracrine mechanism. Vagal sensory afferents, with cell bodies located in the nodose ganglia, provide a direct neuronal link between the gut and the brain. These afferents account for over 90% of the extrinsic sensory innervation of the proximal gut and ~70% of the distal gut (Serlin & Fox, 2020). To determine the necessity of the vagus nerve in mediating peripheral GLP‐1 signalling, a number of different lesioning approaches have been used, including: (1) vagotomy, a surgical approach that involves cutting both subdiaphragmatic vagal trunks; and (2) capsaicin, a chemical administered systemically or directly onto the nerve, that causes necrosis of TRPV1‐expressing neurons. Consistent with a role for the vagus nerve in mediating the effects of peripheral GLP‐1, chemical or surgical lesioning of the vagus nerve increases meal size (Gonzalez & Deutsch, 1981; Mönnikes et al., 1997; Traub et al., 1996; Yamamoto & Sawa, 2000; Yox et al., 1991), postprandial hyperglycaemia, and gastric emptying (I˙meryüz et al., 1997). Crucially, capsaicin or vagotomy abolish peripheral GLP‐1 induced satiation (Abbott et al., 2005; Talsania et al., 2005; Hayes, Kanoski, de Jonghe, et al., 2011; Kanoski et al., 2011; Labouesse et al., 2012), glucose tolerance (Abbott et al., 2005; I˙meryüz et al., 1997) and inhibition of gastric emptying (I˙meryüz et al., 1997). Similarly, the necessity of an intact vagus nerve for mediating all of GLP‐1's effects was demonstrated in human subjects that had undergone vagotomy (Plamboeck et al., 2013). These data suggest that an intact vagus nerve is required to mediate the physiological functions of peripheral GLP‐1. However, a limitation of vagotomy and capsaicin is that they do not provide organ level specificity and do not distinguish the relative importance of afferent and efferent fibres that respectively convey information to, or from, the brain. Recently, the use of a more selective approach for gut‐specific vagal deafferentation, using the neurotoxin saporin conjugated to the gastrointestinal hormone cholecystokinin (CCK‐8) and injected into the nodose ganglia, confirmed that vagal sensory signalling from the gut to the brain is necessary for peripheral GLP‐1 induced satiation (Diepenbroek et al., 2017).

The experiments described above do not distinguish between direct and indirect actions of GLP‐1 on vagal sensory neurons. Direct activation is supported by the expression of GLP‐1 receptor in a subpopulation of vagal sensory neurons (Nakagawa et al., 2004) and increased GLP‐1‐evoked burst firing and cytoplasmic calcium levels in dissociated nodose ganglion neurons (Kakei et al., 2002). *In vivo*, large peripheral injections of GLP‐1 which increase vagal nerve activity are blocked by GLP‐1 antagonists (Bucinskaite et al., 2009). Most convincingly, in a number of animal models, selective removal of GLP‐1 receptors from vagal sensory neurons blocks all of the physiological effects of GLP‐1. The first report used a lentiviral‐mediated RNA interference strategy in rat nodose ganglia to knockdown GLP‐1 receptors by 50% (Krieger et al., 2016). The metabolic phenotype of this partial GLP‐1 receptor knockdown includes impaired glucose control, accelerated rate of gastric emptying, and delayed satiation, without impacting daily food intake. Importantly, knockdown inhibited the effects on eating and gastric emptying induced by peripheral, but not central, injection of GLP‐1. Similarly, viral‐mediated 40% knockdown of GLP‐1 receptors in the left nodose ganglia of rats abolished the satiating effects of endogenous GLP‐1 released in response to oral D‐allulose (Iwasaki et al., 2018). Similar findings were observed using a *Phox2b*‐Cre mouse to generate a more complete genetic deletion of GLP‐1 receptor in a larger number of autonomic and viscerosensory neurons (Sisley et al., 2014; Varin et al., 2019). Thus, GLP‐1 receptor on vagal sensory neurons are necessary to mediate the physiological role of endogenous GLP‐1 released from the gut in response to a meal.

### 2.5 Characterisation of GLP‐1 receptor expressing vagal sensory neurons

In response to a meal, GLP‐1 is released from the basolateral side of L‐cells into the gut mucosa, before entering the lymphatic and portal systems. There is evidence that GLP‐1 can promote firing of vagal afferent fibres innervating the wall of the hepatic portal vein (Nakabayashi et al., 1996), although selective vagotomy of the common hepatic branch had limited impact on the physiological functions of GLP‐1 (Hayes, Kanoski, de Jonghe, et al., 2011). As discussed above, half of gut‐secreted GLP‐1 is already inactivated by the time it reaches the hepatic portal vein, suggesting that GLP‐1 sensing predominantly occurs within the gut mucosa. In support of this idea, vagal sensory fibres abundantly terminate in the intestinal mucosa in close apposition to enteroendocrine cells of the villi (Bohórquez et al., 2015; Richards et al., 2014). Thus, it was surprising to find that *Glp1r*‐expressing vagal sensory neurons do not densely target intestinal villi. Specifically, injection of a Cre‐dependent virus encoding a fluorescent reporter into the nodose ganglia of *Glp1r*‐Cre mice revealed that the majority of *Glp1r*‐expressing vagal sensory neurons densely innervate the stomach, and that innervation decreases rapidly along the intestine (Bai et al., 2019; Williams et al., 2016). Importantly, *Gl* *p1r* *‐expressing* vagal sensory neuron terminals in both the stomach and intestine exhibited a classic mechanoreceptor morphology. *In vivo* calcium imaging of *Glp1r*‐expressing vagal sensory neurons supports a role in sensing gastric and intestinal stretch (Bai et al., 2019; Williams et al., 2016). Furthermore, GLP‐1 was capable of potentiating mechanosensitive vagal nerve responses (Al Helaili et al., 2020). Acute chemogenetic activation of *Glp1r*‐expressing vagal sensory neurons in freely behaving mice reduces food intake for at least 12 h, to an extent sufficient to transiently reduce bodyweight, without any effect on energy expenditure (Brierley et al., 2021). Optogenetic stimulation of *Glp1r*‐expressing vagal sensory neurons in anaesthetised mice increased gastric pressure and resulted in a small reduction in respiration and blood pressure (Bai et al., 2019; Williams et al., 2016). In awake mice, food intake was acutely inhibited in response to optogenetic activation of *Glp1r*‐expressing vagal sensory neuron terminals in the nucleus tractus solitarius (NTS), which also conditioned a modest flavour avoidance (Brierley et al., 2021), but did not elicit an effect in a place preference task (Bai et al., 2019).

**FIGURE 1:**
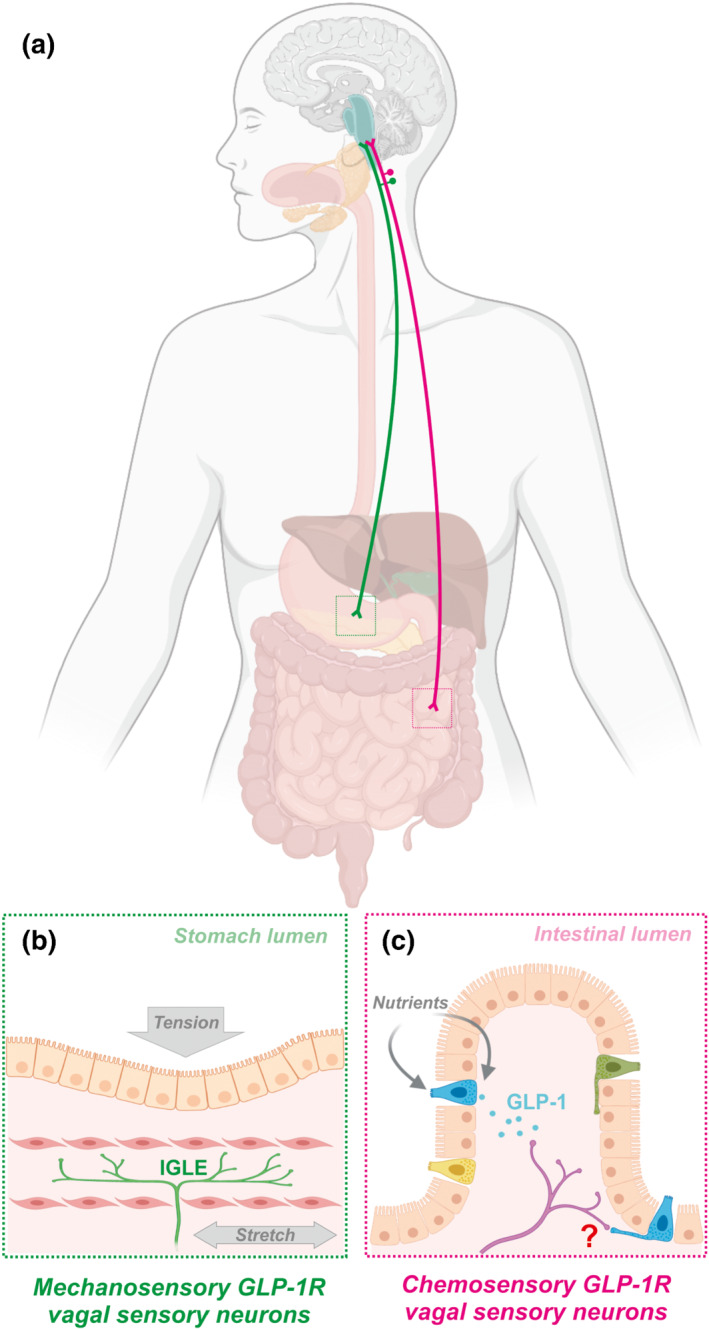
Mechanosensory and chemosensory GLP‐1 receptor (GLP‐1R)‐expressing vagal sensory neuron populations. (a) Vagal sensory neurons which express the GLP‐1 receptor comprises of distinct mechanosensory and chemosensory subpopulations. (b) Mechanosensory neurons predominantly innervate the stomach and detect gastric distension via intraganglionic laminar endings (IGLEs), (c) while chemosensory neurons predominantly innervate the intestinal mucosa and detect GLP‐1 released from enteroendocrine L‐cells. Nutrient sensing by L‐cells occurs primarily via post‐absorptive mechanisms that include receptors and transporters on their basolateral surface, but some nutrients may also be directly sensed via apical projections into the gut lumen. Nutrient detection triggers release of GLP‐1 from L‐cells, which can bind GLP‐1 receptors on local terminals of chemosensory vagal sensory neurons. Direct synaptic signalling between L‐cells and vagal sensory neurons may also occur via neuropod projections present on a subset of L‐cells; however, the physiological role of this signalling pathway remains unclear

A comprehensive transcriptomic study profiling subsets of vagal sensory neurons identified 3 distinct populations of *Glp1r*‐expressing neurons (Bai et al., 2019). In addition to the previously described gastric and intestinal mechanosensitive populations, a separate population was identified, which innervates the mucosal layer of the intestine (Bai et al., 2019; Krieger, 2020). In the intestine, *Glp1r*‐expressing vagal sensory neuron fibres accounted for 6% of the total villous innervation, and only a small fraction of *Glp1r*‐expressing vagal sensory neurons responded to intestinal infusion of nutrients (Williams et al., 2016). This allows for the possibility that endogenous GLP‐1 release from L‐cells activates a subpopulation of GLP‐1 receptor expressing vagal sensory neurons innervating the intestinal mucosa (Figure 1). Defining the function of these separate populations based on their site of innervation using intersectional genetics will be important. Restricting optogenetic stimulation to the mucosa‐innervating *Glp1r*‐expressing population suggests that these neurons alone are not sufficient to suppress eating (Bai et al., 2019) and their role in gastric emptying or glucose homeostasis remains untested. Notably, based on transcriptomic data, a considerable proportion of *Glp1r*‐expressing vagal sensory neurons innervate the hepatic portal vein (Bai et al., 2019; Krieger, 2020). The function of this subpopulation of *Glp1r*‐expressing neurons is unclear, but suggests that GLP‐1 may activate vagal populations upon entering the portal circulation, supporting previous tracing and whole nerve recording studies (Hayes, Kanoski, de Jonghe, et al., 2011).

Blunted GLP‐1 signalling in obesity could provide a partial explanation for increased food intake, increased gastric emptying, and the comorbidity of type 2 diabetes. Although there has been extensive research assessing the impact of obesity on postprandial GLP‐1 release, the results remain inconclusive (Hira et al., 2020). However, there is evidence that the vagal response to GLP‐1 may be blunted. Specifically, the GLP‐1agonist exendin‐4 increased excitability of dissociated nodose ganglion neurons from lean mice, however this was completely absent in cultured neurons from age‐matched obese animals fed a 60% high fat diet for 6 weeks (Al Helaili et al., 2020). Ileal afferent firing in response to GLP‐1 or exendin‐4 were both impaired in obesity, which prevented potentiation of the mechanosensitive response, and was associated with delayed satiation (Al Helaili et al., 2020). Nevertheless, GLP‐1 agonists are highly effective at eliciting weight loss and normalising glucose homeostasis in the obese state. Thus, it remains to be determined to what extent vagal sensory neurons remain sensitive to long acting GLP‐1 agonists and mediate any of their therapeutic effects.

In summary, recent transcriptomic and genetically targeted tracing data have led to a re‐evaluation of the role of GLP‐1 receptor‐expressing vagal sensory neurons. A surprisingly large number of GLP‐1 receptor‐expressing neurons innervate the stomach and are activated in response to gastric distension, an effect that is potentiated by GLP‐1 to suppress eating. Activation of these neurons is blunted in obesity, which may promote hyperphagia in this context. However, a separate population of GLP‐1 receptor‐expressing vagal sensory neurons innervate the intestinal mucosa and are chemosensitive, and have an as yet poorly defined function. Intersectional genetics will be required to delineate the role of these distinct GLP‐1 receptor‐expressing vagal populations and address questions about the exact site of vagal GLP‐1 receptor activation by intestinal and exogenous GLP‐1.

## 3 VAGAL‐DEPENDENT ACTIONS OF GLP‐1 IN THE CNS

While many important questions remain unanswered regarding the mechanisms of peripheral GLP‐1 signalling in the lean and obese states, a robust body of evidence supports a physiological role for vagal‐dependent GLP‐1 signalling in the regulation of eating. However, it is far from clear how these vagal‐dependent peripheral GLP‐1 signals are integrated within the brain to elicit meal termination and/or delay subsequent meal initiation. GLP‐1 is also synthesised and released by neurons in the brainstem, and central GLP‐1 signalling is now strongly implicated in the CNS control of eating behaviour, many aspects of which are regulated by inputs from vagal sensory neurons. In this section we will review what is known and not known about the mechanisms of vagal‐dependent GLP‐1 signalling within the CNS, and in particular the evidence for a vagal link between the peripheral and central GLP‐1 systems.

### 3.1 Central targets of vagal‐dependent peripheral GLP‐1 signalling

In addition to sensory terminals innervating the visceral organs, vagal sensory neurons also send axon terminals to the brainstem dorsal vagal complex. These transmit viscerosensory information via excitatory synapses onto second‐order neurons, principally within the NTS, and to a lesser extent in the adjacent area postrema and dorsal motor nucleus of the vagus (Bai et al., 2019; Garcia‐Luna et al., 2021; Hisadome et al., 2010). Cre‐dependent viral tracing using two independent *Glp1r*‐Cre mouse lines has revealed that the central terminals of *Glp1r*‐expressing vagal sensory neurons innervate the entire rostro‐caudal extent of the NTS, most densely in the medial and commissural subnuclei, and also the area postrema (Bai et al., 2019; Brierley et al., 2021). The composition of the NTS is highly heterogeneous, comprising glutamate, GABA, noradrenaline/adrenaline and glycine containing neurons (Maley, 1996; Travagli & Anselmi, 2016). In addition to these neurotransmitters, NTS neurons synthesise a plethora of peptide neuromodulators, including GLP‐1, cholecystokinin, cocaine‐ and amphetamine‐regulated transcript (CARTPT), corticotropin‐releasing hormone (CRF), galanin, pro‐opiomelanocortin (POMC), enkephalins, somatostatin, and neuropeptide Y (Appleyard et al., 2005; Fekete et al., 2004; Herbert & Saper, 1990; Larsen et al., 1997; Maley, 1996). The NTS has a viscerotopic organisation, that is, it can be anatomically divided into subnuclei based on the origin of its visceral inputs. However, these do not map onto specific neurochemically defined second‐order populations (Travagli & Anselmi, 2016). Based on anatomy alone therefore, vagal‐dependent peripheral GLP‐1 signals could theoretically be integrated by any one or more of the diverse neuronal populations within the dorsal vagal complex.

A plausible and commonly assumed candidate population are the neurons in the caudal NTS which themselves synthesise GLP‐1. These are known as *Gcg* or preproglucagon (PPG) neurons (based on the gene or transcript, respectively) or simply as GLP‐1‐immunoreactive neurons (Gaykema et al., 2017; Hisadome et al., 2010; Merchenthaler et al., 1999; Rinaman, 1999). This putative connection between *Glp1r*‐expressing vagal sensory neurons and PPG neurons in the NTS forms the basis of a prevalent, but largely untested, hypothesis ‐ that peripheral GLP‐1 acts as a satiation/satiety signal by vagal‐dependent activation of the central GLP‐1 system (Grill, 2020; Krieger, 2020; Secher et al., 2014).

### 3.2 Functional interaction of the peripheral and central GLP‐1 systems

While typically thought of as a “gut hormone” produced by L‐cells, GLP‐1 is also produced centrally by PPG neurons via a similar mechanism, i.e. cleavage of proglucagon by prohormone convertase 1/3 (Müller et al., 2019). PPG neurons are predominantly located in the caudal NTS, up to the level of the area postrema, with a contiguous population in the intermediate reticular nucleus (Larsen et al., 1997; Llewellyn‐Smith et al., 2011). As *Glp1r*‐expressing vagal sensory neurons do not terminate ventral to the NTS (Bai et al., 2019; Brierley et al., 2021) and intermediate reticular nucleus neurons involved in eating regulation only receive vagal information indirectly via the NTS (Nakamura et al., 2017), it is unlikely that PPG neurons in the intermediate reticular nucleus integrate vagal signals from peripheral GLP‐1. Hence, this population will not be considered further in this context. By contrast, PPG neurons in the NTS (PPG^NTS^) are anatomically and functionally positioned to be a plausible target of vagal‐dependent peripheral GLP‐1 signalling. PPG^NTS^ neurons project widely throughout the brain, particularly to nuclei in the pons, hypothalamus and forebrain involved in the regulation of eating (Llewellyn‐Smith et al., 2011; McLean et al., 2020; Vrang et al., 2007). The projection pattern of PPG^NTS^ neurons generally mirrors central distribution of the GLP‐1 receptors (Cork et al., 2015; Merchenthaler et al., 1999) and ablation experiments confirm these neurons are the major source of endogenous GLP‐1 in the brain (Holt, Richards, et al., 2019).

The ability of GLP‐1 signalling within the brain to suppress eating is long established (Tang‐Christensen et al., 1996; Turton et al., 1996) and a substantial body of pharmacological evidence has been generated in support of the idea that GLP‐1 is an endogenous, widely acting central neuromodulator of homeostatic and hedonic eating circuits (recently reviewed in detail by McLean et al., 2020; Müller et al., 2019; Trapp and Brierley in this issue). Findings from these pharmacological studies have been considerably extended by recent experiments using transgenic mice expressing Cre under the glucagon promotor, allowing selective interrogation of the functions of PPG neurons (PPG‐Cre and *Gcg*‐Cre lines from Parker et al., 2012 and Gaykema et al., 2017, respectively). The ability of PPG^NTS^ neurons to suppress eating and reduce bodyweight when chemogenetically activated with Gq‐coupled DREADD was first reported by Gaykema et al. (2017). Optogenetic and chemogenetic activation and inhibition experiments in the same mouse line subsequently demonstrated that PPG^NTS^ inputs to corticotrophin‐releasing hormone neurons in the hypothalamic paraventricular nucleus (PVN) were sufficient to suppress eating, and that this effect was independent of glutamate co‐release from these neurons. Importantly, these PPG^NTS^ → corticotrophin‐releasing hormone^PVN^ projections were required for short‐term regulation of eating (Liu et al., 2017).

The physiological roles of PPG^NTS^ neurons in the regulation of eating behaviour, energy expenditure, glucose homeostasis and cardiovascular control were investigated in a series of recent studies using a PPG‐Cre mouse line (Brierley et al., 2021; Holt et al., 2020; Holt, Richards, et al., 2019). Chemogenetic activation experiments replicably confirmed that PPG^NTS^ neurons have the capacity to robustly suppress eating, at a level sufficient to reduce bodyweight, and that this hypophagic effect is not followed by compensatory rebound hyperphagia (Brierley et al., 2021; Holt, Richards, et al., 2019). Eating suppression elicited by chemogenetic PPG^NTS^ activation was not associated with disruption of the behavioural satiety sequence (Brierley et al., 2021), nor conditioning of flavour avoidance (Gaykema et al., 2017), suggesting that these neurons do not mediate the aversive component of stress‐ or visceral malaise‐induced hypophagia. Conversely, PPG^NTS^ neurons are clearly necessary for the hypophagic response to acute restraint stress, as chemogenetic inhibition of these neurons completely abolished stress‐induced eating suppression (Holt, Richards, et al., 2019).

What role the central GLP‐1 system plays in physiological satiation and/or satiety is less clear. Early pharmacological studies observed that the ability of central injections of the GLP‐1 antagonist exendin‐(9–39) to increase acute food intake was dependent on nutritional status (Turton et al., 1996), suggesting central GLP‐1 signalling may not be a “primary” mediator of satiation or satiety. Similarly, no overall effects on food intake or bodyweight were observed following germline knockout of GLP‐1 receptor from the entire CNS (using the *Nes* promotor; Sisley et al., 2014), from hypothalamus, brainstem and enteric nervous system (*Wnt1* promotor; Varin et al., 2019), or from specific hypothalamic neuron populations (*Sim1* and *Pomc* promotors; Burmeister et al., 2016). Conversely, shRNA‐mediated partial knockdown of PPG expression in adult rats transiently increased daily food intake and bodyweight, although effects on meal patterns parameters were not detected and energy expenditure was unaltered (Barrera et al., 2011). In adult mice, sustained elevations in daily food intake and bodyweight were observed following viral‐mediated knockout of GLP‐1 receptors in the PVN (Liu et al., 2017) and following tetanus toxin‐mediated permanent synaptic silencing of *Glp1r*‐expressing neurons in the PVN (Li et al., 2019). This highlights potential species differences within rodent models in this context, and suggests that developmental compensation in germline knockout mouse models may have resulted in an underestimation of the importance of endogenous central GLP‐1 signalling for energy balance.

Further light has recently been shed on this question using acute Gi‐coupled DREADD inhibition and permanent diphtheria toxin subunit A‐mediated ablation of PPG^NTS^ neurons (Brierley et al., 2021; Holt, Richards, et al., 2019). In mice housed and tested under standard condition, that is, with *ad libitum* access to chow diet, neither inhibition nor ablation had any detectable effect on short‐term or daily food intake or bodyweight (Holt, Richards, et al., 2019), nor were any effects observed on meal size, meal frequency, water intake, locomotor activity or energy expenditure (Brierley et al., 2021). However, these studies also tested the effects of these manipulations under experimental conditions designed to promote consumption of 'abnormally' large meals (for *ad libitum* fed laboratory mice), such as refeeding after a moderate fast and time‐limited access to highly palatable liquid diet. Under these conditions, PPG^NTS^ neurons were reproducibly found to be necessary for eating regulation, as both inhibition and ablation caused overeating (Holt, Richards, et al., 2019). Importantly, this overeating was specifically driven by delayed meal termination (Brierley et al., 2021), thereby confirming a physiological role for this neuronal population in the process of satiation. A critical open question is whether the role of PPG neurons in satiation is truly specific to abnormally large meals, or whether, under more naturalistic conditions (characterised by opportunistic feeding on diverse food sources), they play a more important role with relevance to long‐term energy balance.

The finding that PPG^NTS^ neurons are recruited by large food intakes to elicit meal termination strongly suggests that at least some of this population receives vagal signals of gastrointestinal distension, as mechanosensory vagal neurons are crucial mediators of gut‐brain satiation signalling. Several convergent lines of evidence support the idea that PPG^NTS^ neurons receive input from vagal sensory neurons and that they receive signals of gastric distension. Distension of the stomach corpus or fundus by inflation of a gastric balloon is sufficient to induce cFos expression in GLP‐1‐immunoreactive neurons in rats (Vrang et al., 2003), as is consumption of large liquid diet meals (Kreisler et al., 2014; Kreisler & Rinaman, 2016), indicating that these neurons receive direct and/or indirect vagal signals which track meal size. Electrophysiological recordings from PPG^NTS^ neurons in *ex vivo* brainstem slices during electrical stimulation of the solitary tract demonstrated that these neurons receive direct glutamatergic input from the vagal sensory neurons (Hisadome et al., 2010), which has been confirmed using Cre‐dependent monosynaptic retrograde rabies virus tracing from PPG‐Cre neurons (Brierley et al., 2021; Holt, Pomeranz, et al., 2019).

The finding that *Glp1r*‐expressing vagal sensory neurons are predominantly comprised of mechanosensory populations was somewhat surprising, as these neurons had been thought to primarily convey chemosensory information via paracrine signalling of nutrient detection by L‐cells (Bai et al., 2019; Krieger, 2020; Williams et al., 2016). However, considered in light of the evidence that PPG^NTS^ neurons encode satiation and receive vagal distension signals, an updated hypothesis that peripheral and central GLP‐1 systems comprise a single gut‐brain satiation circuit, predominantly driven by mechanical distension signals, is certainly plausible. Evidence for or against such a circuit has, however, been lacking until very recently.

Circumstantial evidence against this hypothesis comes from a recent study investigating the cardiovascular effects of central and peripheral GLP‐1 system manipulations (Holt et al., 2020). In this study, peripheral administration of the GLP‐1 agonist exendin‐4 induced tachycardia in both control mice and mice which had PPG^NTS^ neurons ablated by viral‐mediated diphtheria toxin subunit A expression, and failed to induce cFos in mice with fluorescently labelled PPG^NTS^ neurons. PPG^NTS^ neurons do not themselves express GLP‐1 receptor (Card et al., 2018), but peripheral exendin‐4 might be expected to activate GLP‐1 receptor vagal sensory neurons and their second‐order targets in the NTS. However, current evidence strongly suggests that GLP‐1 receptor vagal sensory neurons predominantly detect gastric distension signals, which may then be amplified by binding of endogenous or exogenous peripheral GLP‐1. Therefore, cardiovascular experiments not designed to specifically test this situation must be interpreted with caution. Nevertheless, the absence of detectable cFos expression in PPG^NTS^ neurons in this experiment provided a hint that this population may not receive input from *Glp1r*‐expressing vagal sensory neurons.

**FIGURE 2:**
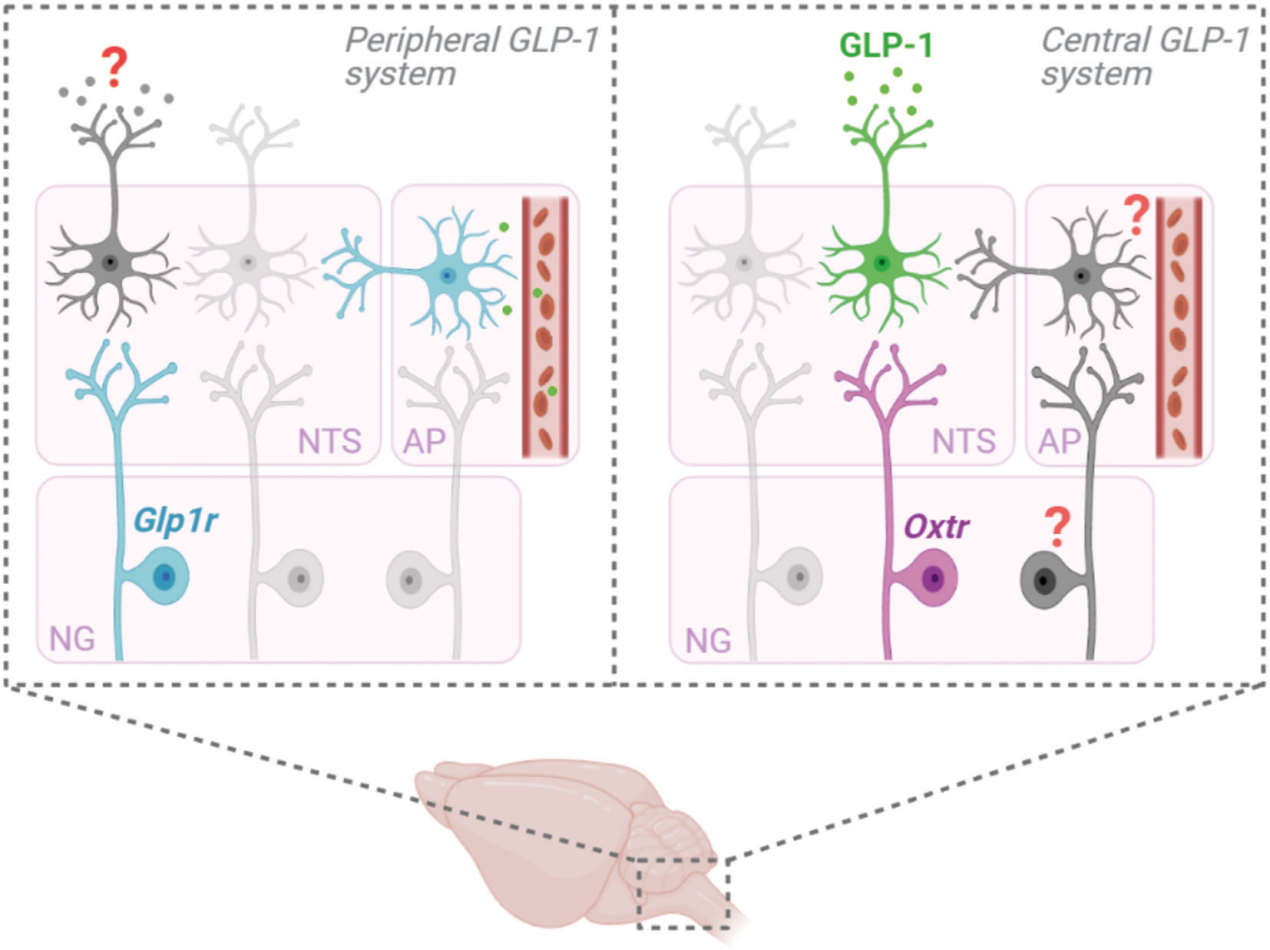
Peripheral and central GLP‐1 system circuitry in the dorsal vagal complex. Schematic overview of circuit connectivity between vagal sensory neuron populations in the nodose ganglia (NG) and their second‐order targets in the nucleus tractus solitarius (NTS) and area postrema (AP) within the peripheral and central GLP‐1 systems. Within the peripheral GLP‐1 system, second‐order targets of GLP‐1 receptor‐expressing (*Glp1r*) vagal sensory neurons have not been identified, but likely include catecholaminergic neurons in the NTS. Similarly, the downstream circuitry of *Glp1r*‐expressing neurons within the AP are poorly characterised. Within the central GLP‐1 system, oxytocin receptor‐expressing (*Oxtr*) vagal sensory neurons provide substantial input to PPG^NTS^ neurons. However, additional vagal populations provide direct input, but these populations have not yet been identified. Furthermore, indirect vagal inputs to the central GLP‐1 system, via neurons in the AP which could integrate hormonal and vagal signals, may offer opportunities for selective pharmacological targeting and warrant urgent investigation

The anatomical and functional connectivity between the peripheral and central GLP‐1 systems has recently been investigated in detail, generating convergent lines of evidence against the idea that there is any substantial vagal (or hormonal) connection between these systems (Brierley et al., 2021). Firstly, chemogenetic and optogenetic activation of *Glp1r*‐expressing vagal sensory neurons only modestly suppressed eating and conditioned avoidance of a paired flavour, suggesting activation of this population induces negative affect. These results contrast to the same manipulation in PPG^NTS^ neurons (Gaykema et al., 2017). Furthermore, while both of these activation strategies induced substantial cFos expression in the NTS *per se*, no significant activation of PPG^NTS^ neurons was detected, overcoming the limitations of the prior experiment using peripheral exendin‐4 administration to test this connection (Holt et al., 2020). Cre‐dependent viral tracing in a double‐transgenic PPG‐yellow fluorescent protein (YFP) x*Glp1r*‐Cre mouse strain determined that the central axon terminals of *Glp1r*‐expressing vagal sensory neurons did not overlap with YFP‐positive PPG neuron somata or dendrites in the NTS. Crucially, quantitative mapping of monosynaptic inputs using rabies tracing and fluorescent *in situ* hybridisation demonstrated that the overwhelming majority of vagal sensory neurons synapsing onto PPG^NTS^ neurons did not express *Glp1r* mRNA (Brierley et al., 2021). However, in this same experiment, a vagal sensory population defined by expression of oxytocin (OT) receptor (*Oxtr*) mRNA was found to provide substantial input to the central GLP‐1 system (Figure 2). OT receptor expression has recently been identified as a marker for a mechanosensory population of vagal sensory neurons that predominantly innervate the small intestine and is largely distinct from the mechanosensory GLP‐1 receptor population, which mostly innervates the stomach (Bai et al., 2019). The functional relevance of this oxytocin input was confirmed by *ex vivo* and *in vivo* experiments showing that application of oxytocin increased calcium transients in PPG^NTS^ neurons, and that these neurons are indispensable for the acute hypophagic effect of peripherally administered oxytocin (Brierley et al., 2021), which is primarily a vagal‐mediated effect (Iwasaki et al., 2015). Conversely, the same diphtheria toxin subunit A ablation approach demonstrated that PPG^NTS^ neurons were completely dispensable for the robust eating suppression and bodyweight loss observed following acute peripheral administration of the GLP‐1 agonists liraglutide and semaglutide. Furthermore, while semaglutide robustly induced cFos expression in the dorsal vagal complex *per se*, increased expression was not observed in PPG^NTS^ neurons. Critically, this study also determined that chemogenetic activation of PPG^NTS^ neurons in mice concurrently administered semaglutide suppressed eating to a greater extent than when semaglutide was administered alone (Brierley et al., 2021).

Overall, recent findings strongly refute the hypothesis that peripheral and central GLP‐1 systems comprise a single, vagal‐mediated satiation circuit. Rather, evidence now suggests the existence of parallel distension‐recruited circuits, respectively comprising: (1) mechanosensitive *Glp1r*‐expressing vagal sensory neurons, acting independently of PPG^NTS^ neurons; and (2) mechanosensitive *Oxtr*‐expressing vagal sensory neurons, driving central GLP‐1 mediated meal termination (Figure 2). This reappraisal of the vagal connectivity between peripheral and central GLP‐1 systems raises a number of further questions, which need to be addressed for a complete understanding of the neurophysiology underlying eating regulation, and to identify new pharmacological approaches for the treatment of obesity.

## 4 OPEN QUESTIONS REGARDING VAGAL‐DEPENDENT REGULATION OF EATING BY GLP‐1

### 4.1 What are the central targets of vagal‐dependent peripheral GLP‐1 signalling?

The neuronal populations in the dorsal vagal complex, which receive input from *Glp1r*‐expressing vagal sensory neurons and integrate signals of gastric distension and peripheral GLP‐1 release, have not been directly identified. Recent data reviewed here do not support a role of PPG^NTS^ neurons in this context, however, alternative candidates considered to date include A2 brainstem catecholamine neurons (Krieger, 2020). Indirect evidence implicates this catecholaminergic neuron population as a potential target, as these neurons are directly innervated by vagal sensory neurons (Appleyard et al., 2007) and express cFos in proportion to the volume of ingested meals (Kreisler et al., 2014) and following intravenous injection of a peripherally restricted GLP‐1 agonist (Yamamoto et al., 2003). Additional candidate populations include NTS proenkephalin neurons, recently identified as targets of a vagal‐dependent gut sugar‐sensing circuit (Tan et al., 2020) and calcitonin (CT) receptor‐expressing neurons, which suppress eating, receive vagal input and are activated by peripheral cholecystokinin and exendin‐4 (Cheng et al., 2020). Cre‐dependent circuit mapping approaches have provided valuable insights into the gut‐brain circuitry mediating GLP‐1 signalling (Bai et al., 2019; Brierley et al., 2021). However, these will need to be combined with intersectional genetic techniques to identify the second‐order targets and ascending circuitry mediating actions of the distinct mechanosensory and chemosensory populations of GLP‐1 receptor vagal sensory neurons.

### 4.2 What is the role of vagal‐dependent oxytocin signalling in central glp‐1 mediated regulation of eating?

That *Oxtr*‐expressing vagal sensory neurons are intestinal mechanosensors which innervate PPG^NTS^ neurons, and that exogenous peripheral oxytocin can suppress eating via central GLP‐1 signalling, are surprising recent findings (Bai et al., 2019; Brierley et al., 2021). Given the considerable therapeutic interest in targeting oxytocin signalling for treatment of obesity and metabolic diseases (Romano et al., 2020), further investigation of the interaction between these signalling system is warranted. The mechanism(s) by which endogenous oxytocin modulates PPG neuron activity, and the physiological and/or pathophysiological conditions under which this circuitry is recruited, remain to be determined. Parvocellular oxytocin neurons project directly to the dorsal vagal complex (Romano et al., 2020) and hence could activate PPG^NTS^ neurons directly and/or indirectly via presynaptic oxytocin receptors on vagal inputs to these neurons. This would be consistent with the ability of exogenous oxytocin to elicit calcium transients in PPG^NTS^ neuron in *ex vivo* brainstem slices (Brierley et al., 2021) and i.c.v. injection of exendin‐(9–39) to block the anorexigenic effect of i.c.v. oxytocin (Rinaman & Rothe, 2002). PPG^NTS^ neurons themselves provide input to oxytocin neurons in the PVN, implying a reciprocal central signalling pathway involving both neuropeptides (Biddinger et al., 2020; Romano et al., 2020), the physiological role of which remains to be determined.

### 4.3 Is vagal activation of central glp‐1 signalling a viable target for obesity pharmacotherapy?

The finding that chemogenetic activation of PPG^NTS^ neurons augments semaglutide‐induced eating suppression (Brierley et al., 2021) suggests that potentiating endogenous central GLP‐1 signalling in combination with GLP‐1R agonist drugs could be a valuable pharmacological strategy for obesity (discussed by Trapp and Brierley in this issue). One way in which this might be achieved is via pharmacological activation of vagal inputs to PPG^NTS^ neurons. Realising the potential of such an approach necessitates comprehensive anatomical and functional characterisation of all vagal populations providing input to PPG^NTS^ neurons, particularly the ~50% of monosynaptic inputs not yet molecularly characterised (Brierley et al., 2021). Particular attention should be paid to the local circuit architecture within the dorsal vagal complex which allows integration of circulating hormonal signals with those conveyed by vagal sensory neurons. As chemosensitive and mechanosensitive vagal sensory neurons terminate in the area postrema (Bai et al., 2019; Brierley et al., 2021; Williams et al., 2016), circulating endogenous and exogenous ligands which cannot pass the blood brain barrier have the potential to modulate gut‐brain vagal signalling at this site. The relevance of this vago‐postremal pathway for regulation of eating is currently unknown and warrants investigation, in particular as this may identify druggable targets (or combinations thereof) for selective manipulation of central GLP‐1 signalling.

### 4.4 Nomenclature of targets and ligands

Key protein targets and ligands in this article are hyperlinked to corresponding entries in the IUPHAR/BPS Guide to PHARMACOLOGY http://www.guidetopharmacology.org and are permanently archived in the Concise Guide to PHARMACOLOGY 2019/20 (Alexander et al., 2019).

## CONFLICT OF INTEREST

The authors have no conflict of interest to declare.

## Data Availability

Data sharing is not applicable to this article because no new data were created or analysed in this study.
